# Human protection drives the emergence of a new coping style in animals

**DOI:** 10.1371/journal.pbio.3001186

**Published:** 2021-04-06

**Authors:** Bastien Sadoul, Daniel T. Blumstein, Sébastien Alfonso, Benjamin Geffroy

**Affiliations:** 1 ESE, Ecology and Ecosystem Health, Institut Agro, INRAE, Rennes, France; 2 Department of Ecology and Evolutionary Biology, University of California, Los Angeles, California, United States of America; 3 COISPA Tecnologia & Ricerca, Stazione Sperimentale per lo Studio delle Risorse del Mare, Bari, Italy; 4 MARBEC, Univ. Montpellier, Ifremer, IRD, CNRS, Palavas-Les-Flots, France

## Abstract

Wild animals face novel environmental threats from human activities that may occur along a gradient of interactions with humans. Recent work has shown that merely living close to humans has major implications for a variety of antipredator traits and physiological responses. Here, we hypothesize that when human presence protects prey from their genuine predators (as sometimes seen in urban areas and at some tourist sites), this predator shield, followed by a process of habituation to humans, decouples commonly associated traits related to coping styles, which results in a new range of phenotypes. Such individuals are characterized by low aggressiveness and physiological stress responses, but have enhanced behavioral plasticity, boldness, and cognitive abilities. We refer to these individuals as “preactive,” because their physiological and behavioral coping style falls outside the classical proactive/reactive coping styles. While there is some support for this new coping style, formal multivariate studies are required to investigate behavioral and physiological responses to anthropogenic activities.

## Abstract 2

This Essay hypothesizes that when human presence protects wild animals from their natural predators, this predator shield decouples commonly associated traits, resulting in a new range of coping phenotypes, dubbed “preactive.”

## Introduction

The ecological importance of intraspecific phenotypic trait variation is gaining increasing attention [1,2]. Recent results demonstrate that intraspecific variation can play an important role in ecosystem services, which is comparable or even stronger than interspecific variation [2]. Personality is the result of intraspecific variation in behavior where within-individual variations overtime are small enough to ensure repeatable relative differences between individuals [3]. By definition, this implies comparisons with other group members across behavioral traits, i.e., an individual is bolder than another in multiple contexts and over time [4]. Boldness, activity, aggressiveness, and sociability/gregariousness are among the most studied personality traits. A behavioral syndrome is seen when there is significant correlation between personality traits over time and across contexts [5,6]. Overall, bolder individuals are more active and more aggressive, explore their environment faster, but are less social [4]. Recent work has identified molecular and endocrinological mechanisms that further distinguish bold, aggressive, and active individuals from their counterparts [4]. The ecological concept of behavioral syndromes relates to the older notion of coping styles [7], which describes individuals on a reactive–proactive continuum based on their physiological and behavioral capacities to respond to a challenge. Reactive animals, those that are relatively shy, less aggressive, and less active, are also, when challenged by a stressor, characterized by high hypothalamic-pituitary-interrenal/adrenal (HPI/A) responses and low sympathetic activity compared to proactive ones [8]. A very large body of literature has also demonstrated that individuals differ in a range of behavioral and physiological traits that are generally associated with being reactive or proactive [7–10], and many of these have ultimate consequences on traits associated with fitness in specific environments [4,11–14].

The Anthropocene is marked by extensive harvesting, environmental pollution, habitat fragmentation, the introduction of exotic species, widespread tourism, urbanization, and climate change [15]. These multiple anthropogenic actions modify the strength and direction of natural selection within a wide range of environments, triggering profound consequences on the behavior and physiology of many species [16,17]. These anthropogenic disturbances have proliferated across the world and are together known as human-induced rapid environmental changes or HIRECs [15]. However, it is important to emphasize that HIRECs do not create a uniform selection pressure [18], with some of them protecting individuals by creating a predator shield, such as seen with tourism, urbanization, captivity, and to a greater extent, domestication [18]. By extirpating predators, or protecting prey from predation risk, humans are acting on an important source of selection pressure. Prior work has shown that predators are a key driver of the structure of behavioral syndromes [19,20]. Yet, much less is known regarding associated physiological traits. Here, we predict that when HIRECs protect prey from their predators, we may see a decoupling of correlated behavioral and physiological traits within an existing coping style, which is then accentuated by evolutionary advantages of being around humans but requires individuals to habituate to their presence. This decoupling would create a new set of correlated traits and hence, a new coping style, that is characterized by animals having both proactive and reactive features.

## Genetic correlations underlying coping styles

Debate remains about evolutionary advantages of interindividual variation in coping styles and the underlying mechanisms maintaining this variation within populations and species. Nevertheless, coping styles are also under selection and can evolve since prior work has shown both that they are heritable and that there are some genetic correlations between behavioral and physiological traits [21,22]. At the genetic level, correlations between behavioral and physiological traits can arise either from linkage disequilibrium or pleiotropy [23]. Linkage disequilibrium is the result of a nonrandom association of alleles at different loci, while pleiotropy recognizes that a locus has multiple effects because it encodes proteins with multiple or cascading effects. Similarly to other genetic correlations, if underlying correlations of coping styles emerge from linkage disequilibrium, then they could rapidly be weakened once selective regimes are relaxed [23]. Multiple studies show limited to absent correlations between physiological and behavioral traits and describe phenotypes that differ from the classical coping styles [24,25]. We therefore suggest that correlations in the wild arise only in specific environments, where trade-offs and constraints force their emergence as a consequence of linkage disequilibrium rather than pleiotropy. Correlational selection, selecting for combinations of traits rather than individual traits, often results in genetic correlations based on linkage disequilibrium [26]. Previous work suggests that this explains the correlations between behavioral and nonbehavioral phenotypes [21,27,28]. Thus, in situations and contexts that reduce or eliminate these constraints, correlations between traits can erode within few generations and favor a new genetic correlation [26].

As with the arrival of any other animal, especially the arrival of top predators, the arrival of humans in an environment can modify the general structure of the ecosystem by modifying trophic and social interactions within and between species [29]. These changes, and the associated new constraints on the environment, are necessarily context dependent but are relevant for all levels of human presence that range from ecotourism to domestication. To persist in this new environment, individuals must cope, and populations must adapt. Coping occurs on relatively short timescales by phenotypic changes via plasticity. Over longer, multigenerational timescales, we expect evolutionary changes. We propose here a general framework based on the most recent literature to explain mechanistically the reasons of the emergence of new coping styles in response to human presence that fall outside the more traditionally understood proactive–reactive continuum.

### Human presence modifies the environment and alters selective forces

In nature, correlational selection is based on the trade-off between advantages and disadvantages being at one end of the coping style continuum or the other. In a variety of situations, this trade-off is rooted in the decision to forage or avoid predation risk. Proactive individuals are generally bolder, explore faster their environment, and spend more time foraging, while reactive individuals are less prone to predation because of their increased shyness. Consequently, proactive individuals are favored when predation risk is low, while reactive individuals are considered favored in a predator-rich environment [30].

Tourism, urbanization, and domestication all share an increased proximity to, and interactions with, humans, but we recognize that these anthropogenic experiences differ in a variety of ways. For instance, populations exposed to tourism might also encounter food provisioning or exposure to novel chemicals (e.g., sun cream for aquatic species). Urbanization creates potentially novel types of habitat and is associated with light, noise, air, and water pollution, as well as novel food items [31]. Captive species might also experience changes in their habitat and, depending upon how they are maintained, modified needs for food. Domestication explicitly selects for specific traits, including human tolerance. Despite this non-exhaustive list of differences between contexts, we recently showed that they all lead to a reduction of antipredator traits (behavioral and physiological) when animals are exposed over multiple generations [17]. Hence, insights gained by more proximate studies of physiological and behavioral responses to domestication can inform these responses to tourism and urbanization [17].

Based on multiple previous studies, we hypothesize that an important part of the reduction of antipredator traits is related to the sudden elimination of predators, a factor that all the above-described contexts share (with a different intensity). Indeed, human presence has been demonstrated to create a predator shield which relaxes selection in all these contexts [18] and reduces the value of some behavioral and physiological traits associated with antipredator responses. Additionally, we know that natural selection favors antipredator phenotypes that efficiently reduce predation risk. But when the risk is low, these energetically costly phenotypes, including vigilance, physiological stress responses to predation, or certain escape capacities, may become too costly to maintain [32–34]. Consequently, the release of constraints related to predation will favor individuals with reduced expression of these traits or favor individuals that have the capacity to quickly respond to these new circumstances through plasticity.

The response to reduced predation seems to come with additional constraints related to the interaction with humans and their associated activities. Human presence generates novel types of direct and indirect selection on some specific traits. These encompass the direct effects of selection for phenotypes related to increased production (e.g., growth) in the case of domestication for food consumption or reduced fear toward humans in animals selected to be human commensals (e.g., habituation). However, apart from these well-known specific selective pressures, the direction and intensity of other selective pressures related to human presence are still unclear, and there is no general framework for predicting animals’ responses to human presence in other contexts. Nevertheless, based on most recent literature, we propose that long-term exposure to extensive tourism and urbanization might ultimately result in similar patterns to those observed under domestication.

### Human presence decouples the link between physiological and behavioral traits

**Fig 1:**
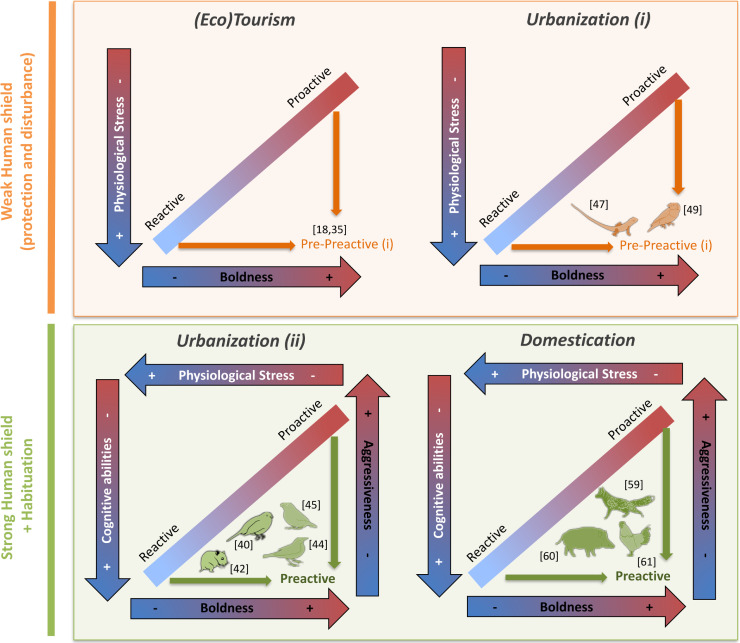
Uncoupling of physiological (in white) and behavioral (in black) coping style traits in, (Eco)tourism, Urbanization, and Domestication contexts. The orange panel represents the case where intensity of human contact is weak, which results in the uncoupling of physiological and behavioral traits within coping styles (i). The green panel represents the cases where the intensity of human contacts is strong, which results in the uncoupling within either physiological or behavioral traits, and creates preactive individuals. The blue to red arrows on the edges represent *x* and *y* axes characterizing behavioral and physiological differences between reactive and proactive coping styles. The smaller arrows (orange or green) highlight the shifts outside the classic reactive–proactive continuum which is related to human presence. Note that we purposely focused on the 2 extreme coping styles to better unpack and illustrate the logic behind our hypothesis. The number refers to the references associated with the finding. *Species design*: *Pierre Lopez (MARBEC)*.

When contact with humans is relatively low but disturbing, high HPI/A reactivity in prey may be associated with increased boldness [35]. This falls outside the classic correlations between physiological and behavioral traits underlying coping styles (i.e., bold animals are supposed to be less stressed). Increased boldness was previously observed in response to tourism and ecotourism (Fig 1. (Eco)Tourism), probably as a consequence of the associated human shield and provisioning [18,36]. However, baseline and post-stress glucocorticoid responses seem to strongly depend on the intensity with which humans interact with animals, with severe disturbance generally increasing glucocorticoid production [35], creating bold but stressed individuals.

Although only few studies have simultaneously investigated behavioral and physiological consequences of urbanization, this context seems to produce behavioral changes similar to animals exposed to tourism, by overall favoring bold individuals [18,37–44] (Fig 1). Concerning the stress responses, results are less consistent, and 2 opposite patterns were described in response to urbanization. Some studies observed a reduced stress load or stress response [40,44–46], while others highlighted increased post-stress corticosterone production [47,48] or increased heart rates [49] in urban animals (Fig 1. Urban (i) and Urban (ii)) [50]. We suppose that these differences are consequences of differences in the level of habituation of species toward humans and propose that some urbanization contexts lead to situations similar to what can be observed in response to tourism (i.e., increased physiological stress; Urban (i)) while animals habituated to humans in a very urbanized context (Urban (ii)) rather respond by a reduction in physiological stress.

### Habituation to humans decouples the link within physiological and behavioral traits

We recognize that many characteristics vary between those studies showing differential stress response, in addition to and including the types and gradients of human interaction. However, we suggest that a valuable way to frame these responses is to view the discrepancy between results being explained by the intensity of human contact. Studies reporting reduced physiological stress [40,44,45] (Fig 1, Urbanization (ii)) generally investigate animals that have been around humans for several generations and have become somewhat commensal, using humans as a source of food or protection. Urbanization may also be associated with selection for increased cognitive abilities [45,51] and behavioral plasticity [39,41,42,52,53]. Although some studies showed no significant associations [54,55], multiple studies showed that urbanization may also increase cranial capacity of some mammals and birds, suggesting that behavioral plasticity is favored either by selection or by differential settlement [56–58]. Hence, long-term exposure to humans results in decreased stress, specific of proactive individuals, and increased cognitive abilities, rather characteristic of reactive individuals (Fig 1, Urbanization (ii)). For behavior too, this long-term association can uncouple boldness and aggressiveness, as recently seen with urban northern cardinals (*Cardinalis cardinalis*) that were bolder, but less aggressive, than rural individuals [44] (Fig 1, Urbanization (ii)), close to what is observed with domestication.

Some of our best knowledge on the physiology and behavior of domesticated species has been gathered in foxes (*Vulpes vulpes*) that have been domesticated for now more than 40 generations [59], domestic guinea pigs (*Cavia porcellus*) [60], and chickens (*Gallus gallus domesticus*) [61]. Selection for tameness decreases fear-related traits [59–61], cortisol production, and associated gene pathways [60–62], which means that tame animals are bolder and more proactive (Fig 1, Domestication). However, domestication also decreases aggressiveness [59,60], which is consistent with them being concurrently more reactive [63]. Increased social cognitive abilities and neurogenesis were also observed multiple times in domesticated animals (Fig 1, Domestication) [60,64–66],and are generally reported to be also correlated with reactive individuals [67]. Nevertheless, it is worth mentioning that these are general responses which might diverge depending on husbandry practices in animals domesticated for intensive production purposes. For instance, inappropriate feeding procedures which limit access to food (e.g., inappropriate delivery systems or low rations) have been observed to increase aggression, through competition for resources [68]. In addition, rearing animals in a closed barren environment leads to behavioral routines reducing cognitive skills [63], in opposition to enriched environments [69]. Finally, brain size reduction, sometimes considered as a marker of reduced behavioral flexibility [70,71], was observed with domestication in some species that were under strong selection for specific traits [72]. But overall, in both domesticated and highly urbanized animals, we see signs of a decoupling between glucocorticoid production and cognitive abilities and/or between aggressiveness and boldness (Fig 1).

### Toward a mechanistic explanation of the involved processes: The emergence of a new coping style

Overall, there is a decoupling of physiological and behavioral traits in wild animals exposed to HIRECs that relax selection pressures on antipredator behavior and increase human contact. It is essential to realize that it is exceedingly difficult to determine whether all these physiological and behavioral adjustments emerge from (I) differential colonization according to an individual’s traits; (II) plasticity of individuals due to human contact; or (III) evolutionary response that selected for specific traits. We suggest that this is likely to depend on the context. One might expect that sudden tourism presence would lead animals already at that site to readily deal with this new “challenge” through plasticity first and evolutionary adaptation after multiple generations. By contrast, provisioning animals by tourists would attract specific phenotypes to colonize and spread around humans. We expect this to happen both within species and across species. The same logic could apply for urban areas. In both cases, animals would have to adapt to human presence, by becoming bolder for those that are shy (due to provisioning and/or human shield) and possibly become more stressed for those that are relatively bold and whom have to deal with this new environment. We thus expect that these environmental alterations can profoundly change the physiology and behavior of animals affected by those HIRECs offering both protection from predators and new food sources.

We suggest a new term to characterize these individuals: They are “preactive” in that they are part proactive and part reactive. Ultimately, we expect strong interactions with humans to drive the evolution toward such preactive individuals. The emergence of this new preactive coping style results from the decoupling of previously associated traits (since variation underlying coping styles has been shown to be heritable) and is expected when interactions with humans are strong. This uncoupling likely occurs in 2 steps.

**Fig 2:**
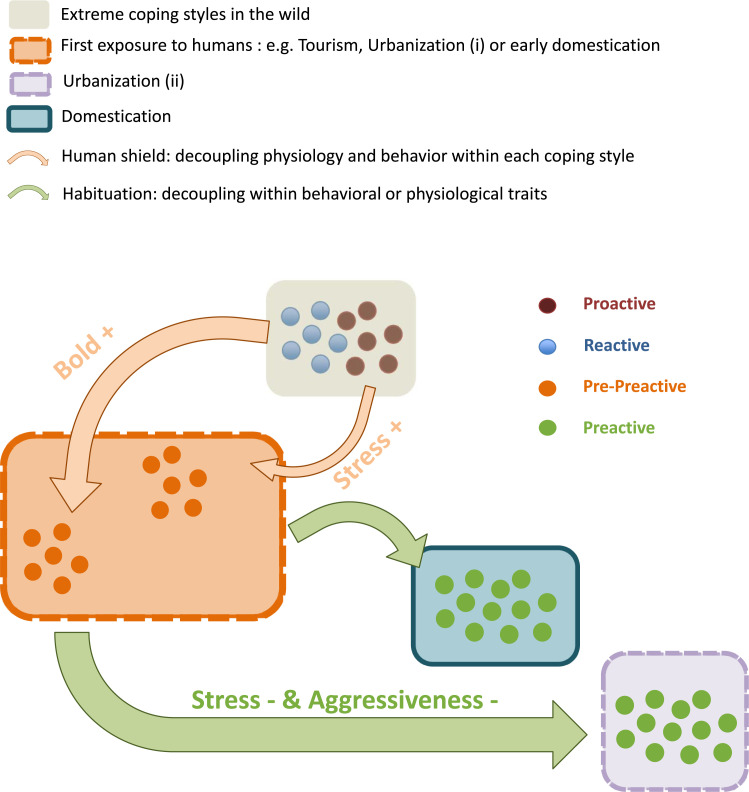
A possible scenario leading to the emergence of preactive individuals. Wild proactive or reactive individuals enter into contact with humans (e.g., via tourism, urbanization, or during the early process of domestication). Reactive animals would become bolder (+), and proactive animals would become more (+) stressed. Then, animals habituate to human presence and become less (−) stressed and less (−) aggressive, resulting in the emergence of preactive animals. Arrow size is proportional to the time necessary for the process to occur. Note that we purposely focused on the 2 extreme coping styles to better unpack and illustrate the logic behind our hypothesis.

First, there is a relaxation of selection by a human shield where behavioral (boldness) and physiological (HPI/A axis) traits are decoupled within each coping style, resulting in pre-preactive (i) animals (Fig 2). As described above, this occurs for animals suddenly exposed to tourism or in early stages of urbanization. Interestingly, this uncoupling was also observed in early steps of domestication of Atlantic salmon (*Salmo salar*), captive-bred populations of a swordtail fish (*Xiphophorus birchmanni*), and captive-bred voles (*Myodes glareolus*) [73–75], confirming that within one or few generations of captivity, a similar and possibly ubiquitous process occurs.

Second, animals associated with humans for longer periods of time habituate to this new situation by decreasing their overall aggression and decreasing their HPI/A reactivity while improving their capacities to cope with environmental perturbations which may be associated with higher neurogenesis and/or neural plasticity (Fig 2).

To further evaluate this proposed cascading process, additional multigenerational and multivariate studies on the behavioral and physiological effects of HIRECs on coping styles are needed. Such studies could help demonstrate how living around humans drives the uncoupling of coping style traits and the emergence of a new consistent coping style. The generality of this concept, and its associated ecological and evolutionary consequences, will become clear only if the underlying genetic basis is understood. All the recent findings suggesting a link between protection from predators, and the emergence of a new coping style leaves another open question: whether this also occurs when domesticator–domesticate relationships involve other nonhuman species since such specific commensalisms exist in the wild [76]. We hope that future studies will allow us to better describe the conditions and the dynamics of the processes that favor the emergence of preactive individuals and help us understand the underlying mechanisms of these changes.
